# A Prospective Study of Children Aged 0–8 Years with CAH and Adrenal Insufficiency Treated with Hydrocortisone Granules

**DOI:** 10.1210/clinem/dgaa626

**Published:** 2020-09-04

**Authors:** Uta Neumann, Katarina Braune, Martin J Whitaker, Susanna Wiegand, Heiko Krude, John Porter, Dena Digweed, Bernard Voet, Richard J M Ross, Oliver Blankenstein

**Affiliations:** 1 Charité Universitaetsmedizin Berlin, Berlin, Germany; 2 Diurnal Ltd., Cardiff, United Kingdom; 3 University of Sheffield, Sheffield, United Kingdom

**Keywords:** congenital adrenal hyperplasia, adrenal insufficiency, hydrocortisone, saliva, children

## Abstract

**Context:**

Children with congenital adrenal hyperplasia (CAH) and adrenal insufficiency (AI) require daily hydrocortisone replacement with accurate dosing.

**Objective:**

Prospective study of efficacy and safety of hydrocortisone granules in children with AI and CAH monitored by 17-OHP (17-hydroxyprogesterone) saliva profiles.

**Methods:**

Seventeen children with CAH (9 male) and 1 with hypopituitarism (male), aged from birth to 6 years, had their hydrocortisone medication changed from pharmacy compounded capsules to hydrocortisone granules. Patients were followed prospectively for 2 years. In children with CAH, the therapy was adjusted by 17-OHP salivary profiles every 3 months. The following parameters were recorded: hydrocortisone dose, height, weight, pubertal status, adverse events, and incidence of adrenal crisis.

**Results:**

The study medication was given thrice daily, and the median duration of treatment (range) was 795 (1–872) days, with 150 follow-up visits. Hydrocortisone doses were changed on 40/150 visits, with 32 based on salivary measurements and 8 on serum 17-OHP levels. The median daily mg/m^2^ hydrocortisone dose (range) at study entry for the different age groups 2–8 years, 1 month to 2 years, <28 days was 11.9 (7.2–15.5), 9.9 (8.6–12.2), and 12.0 (11.1–29.5), respectively, and at end of the study was 10.2 (7.0–14.4), 9.8 (8.9–13.1), and 8.6 (8.2–13.7), respectively. There were no trends for accelerated or reduced growth. No adrenal crises were observed despite 193 treatment-emergent adverse events, which were mainly common childhood illnesses.

**Interpretation:**

This first prospective study of glucocorticoid treatment in children with AI and CAH demonstrates that accurate dosing and monitoring from birth results in hydrocortisone doses at the lower end of the recommended dose range and normal growth, without occurrence of adrenal crises.

The most common cause of adrenal insufficiency (AI) in young children is congenital adrenal hyperplasia (CAH) (1). Patients require lifelong glucocorticoid replacement therapy (2). The recommended therapy in childhood is hydrocortisone given 3 to 4 times daily (2). It is necessary to adjust the glucocorticoid dose in the growing child, and timed hormone measurements, including 17-hydroxyprogesterone (17-OHP), are used to monitor CAH control (2). Successful therapy of AI in childhood requires access to a multidisciplinary team of health care professionals, regular clinic visits, and reliable medication to avoid adverse effects (3). Until 2018, the lowest available licenced preparations of hydrocortisone were 10 mg tablets in Europe and 5 mg tablets in the United States. As scored tablets are licensed to be divided into halves, the lowest possible available dose was 5 mg (Europe) and 2.5 mg (US), respectively. However, these doses are not appropriate to treat neonates, infants, and young children with AI, as they require a daily dose of 10 to 15 mg/m^2^, with single doses as low as 0.5 mg (4). Crushed hydrocortisone tablets suspended in water are often used in some countries (2), though accurate dosing is not possible, as hydrocortisone does not dissolve well in water and may adhere to plastic material when applied with syringes (5, 6). Another common practice in pharmacies is to compound hydrocortisone, which is often mixed with sucrose to overcome the inherent bitterness of hydrocortisone. However, a German study demonstrated that up to 25% of compounded batches do not fulfil the acceptance criteria of the European Pharmacopeia in uniformity of net mass or drug content, or are labeled inaccurately (7). In Europe, hydrocortisone granules have now become licensed for children with AI from birth to 18 years of age and are available in low doses of 0.5, 1, 2, and 5 mg. They were developed to address the age group-specific needs of neonates, infants, and young children (8, 9). As part of the development program, a single dose clinical trial was undertaken in neonates, infants, and children under 6 years of age with AI, the majority of whom had CAH (10). The children were then invited to participate in a prospective follow-up study of continued treatment with hydrocortisone granules. It was not possible to include a control group for the hydrocortisone granules, as there is no licenced formulation providing a hydrocortisone dose below 5 mg and the regulatory authorities recommended against using compounded medication. This manuscript reports the results of this prospective study.

## Methods

### Protocol

A prospective follow-up study with patients who had been recruited from a previous single-dose pharmacokinetic study of hydrocortisone granules was performed over a duration of 2.5 years. The first 3 clinic visits were scheduled monthly, followed by visits every three months (3-monthly). Children who withdrew early were included in baseline and safety analyses but excluded from further analysis.

### Patients

A total of 18 patients that included 10 male, all Caucasian, 1 male with congenital hypopituitarism, and the others with genetically confirmed CAH, were included in the study. All of them successfully completed the previous study with single dosing of the study medication, whose inclusion criteria were age <6 years, confirmed AI (inappropriately low cortisol level), receiving appropriate adrenocortical replacement therapy (hydrocortisone +/- fludrocortisone), adequately hydrated and nourished status at the screening visit, and the ability of the parents/caregivers to understand and give written, informed consent according to the German Medicinal Product Act (AMG §40 3b) (1). Exclusion criteria included: clinical signs of acute infection; fever or current adrenal crisis (although subjects could be re-evaluated for eligibility after acute episodes); inability of the child to take oral therapy; any surgical or medical condition that, in the opinion of the investigator, could have placed the subject at higher risk from his/her participation in the study; and parents/caregivers of subjects unwilling to consent to the saving or propagation of pseudonymized medical data for study reasons. The patients were enrolled into one of 3 cohorts, according to their age at study entry in the previous study (Table 1).

**Table 1.** Patient characteristics at baseline, median (range): classification in 3 cohorts according to age at study entry in previous study

	Cohort 1	Cohort 2	Cohort 3	Overall
Number	n = 9	n = 6	n = 3	n = 18
Female/male	4/5	2/4	2/1	8/10
Age (days)	1316 (1077–2084)	748 (394–923)	46 (36–145)	1000 (36–2084)
Age (months)	43 (35–68)	25 (13–30)	2 (1–5)	33 (1–68)
BMI (kg/m^2^)	16.4 (13.7–22.1)	17.5 (16.4–20.1)	16.0 (13.2–16.8)	16.83 (13.2–22.1)
BSA (m^2^)	0.71 (0.58–0.84)	0.56 (0.44–0.60)	0.27 (0.20–0.33)	0.59 (0.20–0.84)

Cohort 1: 2 to <6 years; cohort 2: 1 month to 2 years; and cohort 3: <28 days.

Abbreviations: BMI, body mass index; BSA, body surface area.

### Treatment

The hydrocortisone dose was given according to the manufacturer’s instructions (Alkindi Summary of Product Characteristics). Children´s hydrocortisone medication was changed from pharmacy compounded capsules to hydrocortisone granules, continuing the same dose. Patients were followed prospectively for 2 years. In children with CAH, therapy was adjusted by 17-OHP salivary profiles timed prior to each dose of one day every three months. Compliance with the intake of hydrocortisone granules was calculated on the basis of the number and strength of capsules dispensed and returned, the number of days between visits, and the daily dose of hydrocortisone prescribed for the time interval.

### Measurements

At each visit, the following measurements were taken: weight, height, blood pressure, heart rate, and a physical examination including pubertal status. In CAH patients, salivary 17-OHP levels were used for dose adjustments in accordance with the clinic’s standard practice, based on at-home sampling every 3 months starting from 3–6 months of age. Patients were trained to chew on a swab (Salivette, Sarstedt, Germany) for at least 3 minutes immediately before each dose of hydrocortisone for 2 consecutive days, that is, 6 samples. In children below 1 year of age, the parents/caregivers wiped the oral cavity with the Salivette swab to collect saliva. Serum measurements of 17-OHP were only made if the child was unable to provide a 17-OHP saliva profile or when renin levels were measured annually as part of routine clinical care. Blood sampling was not timed prior to hydrocortisone intake, as it was performed when children visited the outpatient clinic. Adverse events following the first intake of study medication, including stress dosing, were recorded at every visit.

### Calculations of weight and height standard deviation scores, and parental target height standard deviation score

Weight and height standard deviation (SD) scores were based on German KIGGS-Data (11). The parental target height was calculated using the method of Hermanussen/Cole (12).

### Methods of hormone measurements

Hormones were measured using the clinic’s standard practices. Serum 17-OHP was measured using the IDS-iSYS Multi-Discipline Automated System 17-OHP assay (Immunodiagnostic Systems Limited, Tyne & Wear, United Kingdom) with a lower limit of quantification (LLOQ) 0.31 ng/mL. Salivary 17-OHP was measured using the Demeditec free 17-OHP in saliva ELISA kit (Demeditec Diagnostics GmbH, Kiel, Germany). Both measurements were done according to the manufacturer’s instructions. Performance parameters for the assays (serum/saliva) are intra-assay variability: 2.4%/5.2%; interassay variability: 6.2%/5.3%; and LLOQ: 0.3 ng/ml/11.3 pg/mL.

### Hydrocortisone dosing and dose titration

Hydrocortisone dose in newborn babies was determined by the treatment center. This was 2 mg hydrocortisone thrice daily for the main center, while the 2 newborns from satellite centers were treated with 2-1-1 mg and 1-1-1 mg daily, respectively. Initially, the dose was adjusted by serum 17-OHP, reducing the dose as soon as serum 17-OHP was normalized to a minimum of 2-1-1 mg in 2 patients and 1-1-1 mg in the third newborn baby. From about 6 months of age, dosing of hydrocortisone was adjusted using timed saliva 17-OHP profiles in patients at the central clinic, while the patients from satellite centers were monitored by serum 17-OHP. The central clinic has established daytime specific target ranges for predose salivary 17-OHP based on the reference range for healthy children (3.0–32.9 pg/mL). Target ranges for CAH patients were defined as follows:

- Between 2:00 am and 10:00 am: <2.5-fold of the upper reference limit was considered “overtreatment,” 2.5- to 5-fold was considered “good disease control,” 5- to 10-fold was considered “acceptable,” and >10-fold levels were considered “undertreatment.”
- Between 10:00 am and 2:00 am the ranges were reduced by 50%, as follows: <1.25-fold of the upper reference limit was considered “overtreatment,” 1.25- to 2.5-fold was considered “good disease control,” 2.5- to 5-fold was considered “acceptable,” and >5-fold levels were considered “undertreatment.”

Hydrocortisone doses were adjusted according to the predose 17-OHP salivary profiles. If the value for 17-OHP was out of range, the dose 8 hours before that sample time point was adjusted. Doses were adjusted by the smallest increment possible, usually 0.5 to 1.0 mg, depending on how close the current daily hydrocortisone dose was to the target range of 10 mg/m^2^/day. Hydrocortisone dose in mg per body surface area was always calculated but was not the sole basis for dose adjustment.

### Sick day rules, adrenal crisis

Parents/caregivers were trained in using the clinic’s standard sick day rules for children with AI in case of fever and illness, and increased their daily hydrocortisone dose according to local guidelines: for fever >38°C, a double hydrocortisone dose; for fever >39°C, a triple dose; and for fever >40°C, a 5-fold dose. A repeated full dose was recommended in case of vomiting, and a prednisolone suppository (100 mg) and urgent presentation to a doctor was indicated when the vomiting continued. Adrenal crisis was defined as a profound impairment of general health and at least 2 of the following conditions: hypotension (systolic blood pressure <100 mmHg), nausea or vomiting, severe fatigue, hyponatremia, hypoglycemia, and hyperkalemia (13).

### Ethics

The study was conducted in accordance with the ethical principles in the Declaration of Helsinki (1996) and in accordance with International Council for Harmonisation Good Clinical Practice and the Independent Ethics Committee (study number at the local ethics committee 15/0375 – EK 15) and BfArM (the German regulatory authority) requirements. All parents/caregivers gave their written informed consent and all children >3 years of age were informed separately about the study procedures. Subjects remained enrolled in the study unless they met the study withdrawal criteria or until hydrocortisone granules became commercially available.

## Results

### Patients and visits

Eighteen patients were recruited. In total, 6 patients withdrew during the study: 4 withdrew within the first month and their data were only included in the baseline and safety analysis; 1 patient withdrew after 5 months and has data included but did not attend the final visit, and the growth charts therefore exclude this patient; and 1 patient withdrew before first dosing, resulting in inclusion of only demographic data. One patient also withdrew before dosing, but subsequently re-enrolled, and the respective data are included from that date onwards. One male patient with hypopituitarism was only included in the demographic data and safety data analysis, as growth was impaired by the underlying condition. Seventeen patients (8 females, 9 males), with genetically confirmed classic CAH, had salt wasting CAH and were additionally treated with fludrocortisone. All withdrawals were within the older patient cohorts 1 (n = 5) and 2 (n = 1) and were related to difficulties adapting from sweetened pharmacy-compounded powder to tasteless dry granules for the administration of the nighttime dose while sleeping. A protocol amendment was made during the study to allow for the use of soft food for administration of the granules. Study follow-up was up to 2.5 years: median (range) 795 days (1–872 days). Of the 12/17 children with CAH who received hydrocortisone granules for more than 1 month, the mean number of visits/patient (SD) was 10.7 (4.93), with a median (range) of 13 visits (1–15). Overall, 150 follow-up visits were analyzed. The compliance of patients included in the study until their withdrawal or until the end of the study was good, with a median of 98.9% of treatment days with the correct intake of study medication.

### Growth

**Figure 1.**
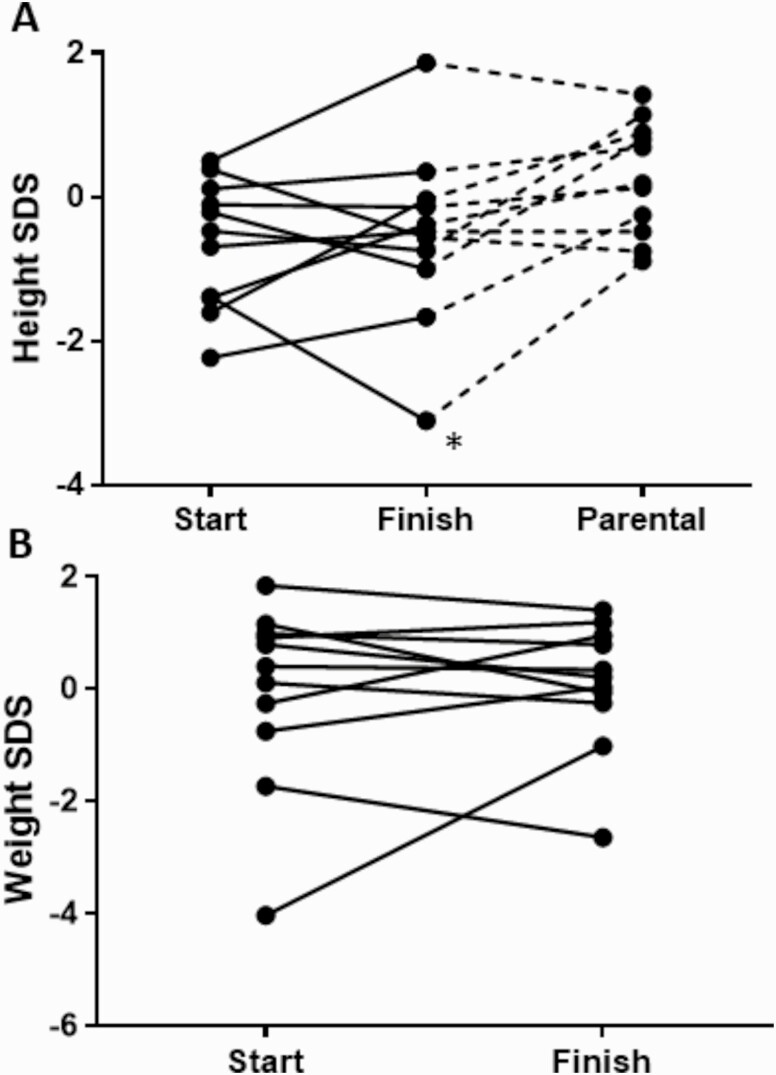
Height and weight of individual patients at start and finish of study in patients that had more than 6 months of treatment (n = 11): **A:** Height standard deviation score (SDS) compared to parental target height. **B:** Weight SDS. *This child had renal hypoplasia in addition to CAH.

Standard deviation scores for height and weight in the 11 children with CAH treated with hydrocortisone granules for over 6 months were evaluated over the study period, and all but 1 showed normal growth with no trends for accelerated or reduced growth. The 1 patient with reduced growth had congenital renal hypoplasia in addition to CAH, which explained the poor growth. When adjusted for target height, two-thirds of the children converged towards their expected height centile (Fig. 1A). The mean difference between z-scores of actual height and target height (SD) decreased from 1.04 (0.71) to 0.89 (0.72). The z-scores for weight and BMI decreased toward the 50^th^ percentile in half of the patients (Fig. 1B).

### Safety and sick day rules

There were no deaths, no severe treatment-emergent adverse events (TEAEs), no TEAEs leading to withdrawal from the study, and no TEAEs with a suspected causal relationship to hydrocortisone granules. No cases of adrenal crisis were reported. Nine severe adverse events were reported in 3 patients (gastroenteritis, vomiting, urinary tract infection, erysipelas), all considered unrelated to hydrocortisone granules. A total of 193 TEAEs were reported by 14 subjects (77.8%), with the most commonly occurring of which were pyrexia, n = 45 in 10 patients (45/10), gastroenteritis (15/9), viral upper respiratory tract infection (21/7), and vomiting (14/7). In 172/193 treatment-emergent adverse events (13 subjects), and in all 9 severe adverse events, the hydrocortisone dose was increased using the sick day rules, and of these TEAEs 42 were reported by 1 subject (Table 2). The most common causes of sick day episodes were fever, vomiting or diarrhea, respiratory tract infections, or other (most commonly stress or excitement related symptoms or abdominal pain). In the subject with 42 episodes of implementing sick day rules, further investigation found that the parents were dosing for excitement and nonspecific abdominal pain on multiple occasions, for example, at a birthday party or New Year’s Eve. With the majority of TEAEs, the hydrocortisone dose was increased according to the sick day rules; in 7 cases the increased stress dose was given for 7 to 12 days, and in all other events the duration was less than 7 days.

**Table 2.** Implementation of sick day rules

Cause	Episodes	Subjects	Percentage of Subjects Overall^*c*^
Vomiting	14	5	28
Fever	101	13	72
Diarrhea	9	2	11
Other^*b*^	47	10	56
No reason given	1	1	6
Total^*a*^	172	13	72

^*a*^ 42 episodes in 1 individual.

^*b*^ Other causes are: Excitement/stress (birthday party, New Year´s Eve, Sleepover in kindergarten, first day at school), Abdominal pain, Infection, Injury (e.g., laceration, injury of teeth after fall), Headache, Exercise, Operation/Procedure, Rash

^*c*^ The percentage of subjects overall was calculated from the overall subject number n = 18.

### Hydrocortisone dose

The hydrocortisone dose was within the recommended dose for body surface area over time, and at the lower end of the recommended treatment ranges for CAH at the end of the study (Table 3). Doses used at different time points varied from 0.5 to 10 mg, and the dose distribution varied from the beginning to the end of the study within age groups, with the older patients having a greater proportion of their dose in the morning and nighttime (Table 4).

**Table 3.** Daily dose of hydrocortisone and fludrocortisone at the beginning and end of study in different age groups, median (range), and number

Visit	Cohort 1	Cohort 2	Cohort 3	Overall
First visit				
HC dose (mg/m^2^/day), median (range)	11.9 (7.20–15.5)	9.9 (8.6–12.2)	12.0 (11.1–29.5)	11.4 (7.2–29.5)
N	9	4	3	16
FC dose (µg/m^2^/day), median (range)	102 (62–159)	146 (124–180)	492 (369–900)	127 (62–900)
N	9	4	3	16
Last visit				
HC dose (mg/m^2^/day), median (range)	10.2 (7.0–14.4)	9.8 (8.9–13.1)	8.6 (8.2–13.7)	9.7 (7.0–14.4)
N	4	4	3	11
FC dose (µg/m^2^/day), median (range)	61 (45–77)	70 (65–94)	213 (92–216)	72 (45–216)
N	4	4	3	11

The 3 cohorts were classified according to age at study entry in a previous study. Cohort 1: 2 to <6 years; cohort 2: 1 month to 2 years; and cohort 3: <28 days.

Abbreviations: FC, fludrocortisone; HC, hydrocortisone; N, number.

**Table 4.** Average dose and dose distribution of hydrocortisone (morning – afternoon – nighttime dose) across visits according to the age of the children

	Average Dose Distribution (%)	Mean HC Dose (mg)	Proportion of Administrations Containing 0.5 mg Granule Dose (%)
Children aged 0–3 months	33.3 – 33.3 – 33.3	1.5 – 1.5 – 1.5	0 – 0 – 0^*a*^
Children aged 4 months to <2 years	46 – 22 – 32	2.3 – 1.1 – 1.6	0 – 7 – 10
Children aged 2 to <4 years	40.6 – 18.7 – 40.6	2.6 – 1.2 – 2.6	41 – 18 – 23
Children aged 4 to 8.4 years	36 – 14 – 50	3.2 – 1.3 – 4.6	18 – 43 – 15

Abbreviation: HC, hydrocortisone.

^*a*^ Half of the patients were treated with a hydrocortisone dose of 2-2-2 mg and half of the patients were treated with 1-1-1 mg, resulting in a mean dose of 1.5-1.5-1.5 mg.

### Fludrocortisone dose

Daily fludrocortisone dose was divided into 1 to 3 single doses per day and adjusted according to the renin level (Table 3).

### Monitoring by saliva and blood sampling

17-OHP salivary profiles were collected at home prior to 82 of the 150 investigated visits (82/150; 54%). Of these 82 investigated 17-OHP salivary profiles, the samples at all three daily dosing times were in range in 44% (36/82), the samples at 2 time points were in range in 28% (23/82), and at only 1 time point in 16% (13/82). In 12% (10/82), all three samples of the whole day 17-OHP profile were out of range. Hydrocortisone doses were changed in 40/150 visits, in 32 visits based on the results of salivary measurements collected at home before the visit, and in a further 8 visits due to serum 17-OHP levels. Of these 40 visits with change of hydrocortisone dose, in 21 visits a single dose was changed, in 13 visits 2 doses were changed, and in 6 visits all 3 doses of a day were changed, amounting to 65 dose changes in total. In 82%, the morning or nighttime dose was changed, resulting in an altered dose distribution across the day, with the lowering of the midday dose. In 12/32 cases, where the dose was adapted based on 17-OHP saliva sampling, a repeat sampling was done within 4 weeks, and in 8/12 cases these control samples were in range.

## Discussion

We have prospectively studied a group of children with AI from 0 to nearly 8 years of age treated with hydrocortisone granules for a total duration of up to 2.5 years. To our knowledge, this is the largest prospective interventional study to date in neonates, infants, and young children with AI. Individualized treatment by titration in these patients was in line with the treating center’s normal practice. The availability of a lowest unit dose of 0.5 mg allowed the doses to range from 0.5 to 10 mg at individual time points during the day. In 21.3% of all administrations, 0.5 mg doses were used. The daily dose per body surface area at the end of the study period across the patient group was at the lower end of that recommended for the treatment of CAH, and it was similar to that recommended for adrenal replacement therapy. Despite this relatively low dose, disease control remained good, as demonstrated by normal growth and the lack of pubertal development. The children experienced common childhood illnesses, resulting in a large number of adverse events reported, none of which were considered related to the study medication, and no adrenal crises occurred.

Recommended hydrocortisone dosing for children with CAH is 10 to 15 mg/m^2^/day, given 3 times daily, although cohort studies show higher doses generally being used in children (14–16). In this study, accurate dosing with hydrocortisone granules using dose changes down to 0.5 mg allowed for dosing at the lower end of the recommended dose range. The thrice daily, individualized, 8-hour dosing regimen used in this study has been used for many years in the main center. While it appears clear that twice daily dosing is less effective in the attainment of physiological cortisol concentrations than thrice daily dosing, no evidence to date has shown a benefit for differently timed regimens for hydrocortisone delivery (14). Pharmacokinetic studies of hydrocortisone in children with AI reveal significant times of high and low cortisol exposure, with frequently very low cortisol levels between dosing (17); therefore, this was also likely in this study.

This is the first prospective study in young children of stress dosing and adrenal crisis and demonstrates that children with AI under 8 years of age encountered multiple intercurrent illnesses; however, despite the hydrocortisone dose being towards the lower end of the recommended dose range, no adrenal crises were seen during this period. Congenital adrenal hyperplasia has been associated with increased risk of mortality. In a cohort of 588 patients with CAH from the Swedish national registry, adrenal crisis has been reported as the leading cause of death followed by cardiovascular disease, which is the leading cause of death in adults globally (18). Prevention of adrenal crises in CAH patients is 1 of the key elements of appropriate glucocorticoid dosing under normal and stress-related situations (14), and the main risk of lowering doses of steroid in conditions with AI is the possible increase in life threatening adrenal crises. A long-term study of adrenal crises in patients with CAH found that receiving lower hydrocortisone doses had associated higher rates of stress dosing and illnesses and were a risk factor for adrenal crises (19). Patients with CAH and AI and their caregivers are taught sick day rules (20), and they are recommended to increase their glucocorticoid intake by doubling, tripling, or 5-fold, increasing the dose whenever they feel unwell with a fever or flu-like illness. In case of vomiting, diarrhea, or severe worsening of illness a prednisolone suppository should be used or an injection of hydrocortisone is recommended. There is no universally accepted regimen of sick day rules. The protocol used in this study, according to the German CAH guidelines, has been successfully used by the main study center (Charité Universitaetsmedizin Berlin) for several years, and it worked well for the study cohort. Up until recently, very little information on the underlying causes that prompt parents to institute stress dosing have been assessed systematically. A retrospective study of El-Maouche et al found an increased number of illnesses and necessary stress dosing in children compared with adults, with the highest rates found in younger children, in particular (19). The follow-up period of our study allowed us to gather such data, with adverse events and stress dosing discussed during the regular visits every 3 months, and it was reassuring that in most cases the stress dosing was being used for physiological stresses. However, 1 patient in this cohort had an excess number of events treated with stress dosing and higher doses were administered, for example, for excitement and nonspecific abdominal pain on multiple occasions. We believe that gathering data on stress dosing can help pediatric endocrinologists to identify families where stress dosing is being used more frequently than expected and where counseling and support can be instituted to avoid the child being exposed to excessive steroid doses.

The drop-out rate in the study can be explained by 2 major factors, depending on age of the children. In neonates, patients were recruited from all over Germany and parents were not willing to travel the long distance to the study center in the follow-up study. In children >2 years of age, those withdrawing generally did not accept the change to a nonsweetened hydrocortisone medication, and so withdrew early. In the remaining study cohort, compliance was high, with a median of >98% of treatment days where study medication was administered as recommended, and this might reflect the clinic’s approach of patient empowerment and education of the patients, parents, and caregivers.

Therapy was predominantly monitored by salivary profiles. If parents provided a child’s salivary 17-OHP profile before the clinic visit, we did not perform serum 17-OHP. However, at the yearly blood sample to check for renin values, we always measured serum testosterone and 17-OHP. These blood samples are not timed and, therefore, in our experience, of limited value as sometimes the serum 17-OHP is increased despite the 17-OHP home salivary profile 2 days before being in range. This might be due to blood sampling inducing an increase in 17-OHP. Additionally, it is difficult to adjust individual doses during the day according to a single serum 17-OHP measurement. When dosing was adjusted by 17-OHP salivary profile, the hydrocortisone dose before each sampling time point was evaluated and changed as required. When dosing was adapted by the serum 17-OHP measurement, the overall dose was adjusted according to the international guidelines by calculating the dose by 10 to 15 mg/m^2^ body surface area divided into 3 single doses (14). The monitoring of CAH is an area where little data exists to provide practice guidelines. According to our experience, salivary sampling in young children worked well, with salivary profiles available at more than half of the clinic visits, so that blood sampling could be minimized. Monitoring by 17-OHP salivary profiles was started at 3–6 months of age. An advantage of 17-OHP saliva sampling is that the collection can be performed at home without further stress for the children, and it allows for individual titration of therapy for each patient. However, more research is required to develop best practice guidance on monitoring in CAH, with our data suggesting that salivary sampling can be considered as a viable alternative to blood sampling.

Based on our findings from this study, which is the largest interventional study in the orphan condition of childhood AI to date, the use of hydrocortisone granules resulted in effective treatment of the children’s AI, as demonstrated by the absence of adrenal crises and a normal growth profile. No safety issues were detected. Over the course of 2.5 years, the patients’ doses of hydrocortisone remained stable and within the recommended range, and they decreased at the end of the study to the lower end of the recommended treatment range for CAH. In conclusion, hydrocortisone granules are an effective treatment for childhood AI, providing the ability to accurately prescribe pediatric-appropriate doses.

## Data Availability

The datasets generated during and/or analyzed during the current study are not publicly available but are available from the corresponding author on reasonable request.

## Additional Information

***Disclosure Summary:*** R.J.M.R. and M.J.W. are Directors of Diurnal Ltd., J.P. is an employee of and B.V. and D.D. are consultants to Diurnal Ltd. U.N. and O.B. report a fee for presentation, and U.N. and K.B. received grants from Diurnal Ltd.
